# Effects of perioperative glycemic indicators on outcomes of endovascular treatment for vertebrobasilar artery occlusion

**DOI:** 10.3389/fendo.2022.1000030

**Published:** 2022-10-05

**Authors:** Mengmeng Gu, Jin Fan, Pengfei Xu, Lulu Xiao, Jinjing Wang, Min Li, Chaolai Liu, Genpei Luo, Qiankun Cai, Dezhi Liu, Lichao Ye, Junshan Zhou, Wen Sun

**Affiliations:** ^1^ Department of Neurology, Nanjing First Hospital, Nanjing Medical University, Nanjing, China; ^2^ Department of Neurology, the General Hospital of Western Theater Command, Chengdu, China; ^3^ Stroke Center & Department of Neurology, the First Affiliated Hospital of University of Science and Technology of China (USTC), Division of Life Sciences and Medicine, University of Science and Technology of China, Hefei, China; ^4^ Department of Neurology, Affiliated Jinling Hospital, Medical School of Nanjing University, Nanjing, China; ^5^ Department of Neurology, Jiangsu Province Hospital of Chinese Medicine, Affiliated Hospital of Nanjing University of Chinese Medicine, Nanjing, China; ^6^ Department of Neurology, The First People’s Hospital of Jining, Jining, China; ^7^ Department of Neurology, Dongguan People’s Hospital, Dongguan, China; ^8^ Department of Neurology, The Second Affiliated Hospital of Fujian Medical University, Quanzhou, China; ^9^ Department of Neurology, Shuguang Hospital Affiliated to Shanghai University of Traditional Chinese Medicine, Shanghai, China

**Keywords:** admission blood glucose, stress hyperglycemia, glycemic variability, endovascular treatment, vertebrobasilar artery occlusions, prognosis

## Abstract

**Objective:**

Endovascular treatment (EVT) is, to date, the most promising treatment of vertebrobasilar artery occlusion (VBAO). The study aimed to determine the influence of perioperative glucose levels on clinical outcomes in patients with acute VBAO treated with EVT.

**Methods:**

We retrospectively collected consecutive VBAO patients received EVT in 21 stroke centers in China. The associations between perioperative glycemic indicators (including fasting blood glucose[FBG], admission hyperglycemia, stress hyperglycemia ratio [SHR] and short-term glycemic variability [GV]) and various clinical outcomes were analyzed in all patients and subgroups stratified by diabetes mellitus (DM).

**Results:**

A total of 569 patients were enrolled. Admission hyperglycemia significantly correlated with increased risk of symptomatic intracranial hemorrhage (sICH) (odds ratio [OR] 3.24, 95% confidence interval [CI]: 1.40-7.46), poor functional outcomes at 90 days (OR 1.91, 95%CI: 1.15-3.18) and 1 year (OR 1.96, 95%CI: 1.20-3.22). Similar significant correlations exist between FBG, SHR, GV and all the adverse outcomes except higher levels GV was not associated with increased risk of sICH (OR 1.04, 95% CI: 0.97-1.12). Subgroup analyses showed that admission hyperglycemia, FBG and SHR were significantly associated with adverse outcomes in non-diabetic patients, but not in DM patients. While, GV was associated with poor functional outcomes regardless of diabetes history.

**Conclusions:**

Admission hyperglycemia, FBG, SHR and short-term GV in VBAO patients treated with EVT were associated with adverse outcomes. The results suggested that comprehensive evaluation and appropriate management of perioperative glucose might be important for patients with VBAO and treatment with EVT.

## Introduction

Vertebrobasilar artery occlusion (VBAO) accounts for 10% to 20% of all large vessel occlusions (1). It could be devastating, resulting in severe disability and death in almost 80% of patients (2). Endovascular treatment (EVT) is currently the most effective method for recanalization of occluded large vessels. However, even if EVT treatment increased the rate of successful recanalization, the prognosis of VBAO patients has not been significantly improved (2, 3).

Many glycemic indicators, such as admission blood glucose (4), fasting blood glucose (FBG) (5) and stress hyperglycemia (6), have inconsistently been reported to be associated with adverse outcomes in patients with anterior circulation stroke (ACS) treated with EVT (7). Furthermore, the postoperative period can be extremely dangerous for patients, especially as the glycemic variability (GV) is associated with adverse clinical outcomes (8). Previous studies have suggested that admission blood glucose of patients with posterior circulation stroke (PCS) is higher than that of patients with ACS (9, 10). Considering that hyperglycemia may aggravate the oxidative stress injury induced by cerebral ischemic reperfusion injury and lead to greater neurological impairment (11, 12), we hypothesized that the prognosis of patients with PCS treated with EVT is more likely to be affected by blood glucose levels.

Using the acute PostErior ciRculation iSchemIc Stroke regisTry (PERSIST, ChiCTR2000033211) data, we aimed to investigate the comprehensive perspective of the relationships between perioperative glycemic indicators and outcomes of patients with PCS treated with EVT, and whether the impact of blood glucose on the incidence of outcomes differed between diabetic subgroups.

## Methods

### Patients

The PERSIST is a retrospective registered trial conducted in 21 stroke centers in China from December 2015 to December 2018, which consecutively collected patients with acute VBAO received EVT treatment. Inclusion and exclusion criteria have been described in detail in previous studies (13, 14). The inclusion criteria were as follows: 1) aged 18 years or older; 2) with acute symptomatic VBAO confirmed by imaging examination (including computed tomography angiography, magnetic resonance angiography or digital subtraction angiography); 3) treated with EVT within 24 hours (estimated occlusion to groin puncture time). The exclusion criteria were: 1) combined with anterior circulation stroke; 2) accompanied by aneurysm or arteriovenous malformation; 3) with mRS score >2 before stroke; 4) participated in any clinical trials; 4) pregnant or breastfeeding; and 5) with incomplete critical baseline data. In this analysis, we further excluded patients who had hemoglobin disorders (i.e. thalassemia), recent blood transfusion, severe hepatic disease and other factors that affected hemoglobin A1c (HbA1c) measurements.

The study was approved by the ethics committee of the First Affiliated Hospital of University of Science and Technology of China (approval number: 2020KY-40). Due to its retrospective nature, patient consent was waived.

### Baseline characteristics

Baseline demographics, medical histories, stroke severity evaluated by the National Institutes of Health Stroke Scale (NIHSS) score, Glasgow Coma Scale (GCS) score, stroke etiology classified according to the Trial of Org 10172 in Acute Stroke Treatment (TOAST) criteria, onset patterns, treatment with intravenous thrombolysis (IVT), estimated occlusion to groin puncture time, puncture to reperfusion time and treatment profiles of EVT were retrospectively obtained by reviewing medical records. Two neuroradiologists, who were unaware of the clinical data and outcomes, retrospectively evaluated all neuroimaging data, including the posterior circulation-Alberta Stroke Program Early CT Score (pc-ASPECTS) (15), the Basilar Artery on Computed Tomography Angiography (BATMAN) score (16), and the American Society of Interventional and Therapeutic Neuroradiology/Society of Interventional Radiology (ASITN/SIR) collateral score (17). Successful recanalization was defined as modified Thrombolysis in Cerebral Infarction (mTICI) score 2b or 3.

### Perioperative glucose levels and short-term GV

We collected available admission blood glucose, fasting blood glucose (FBG) within 24 hours of EVT and first-measured HbA1c. Previous diabetes mellitus (DM) was defined as a known history of DM on admission or the presence of background hyperglycemia (HbA1c ≥ 6.5% [48 mmol/mol]) (18). According to the previous studies (4), admission hyperglycemia was defined as admission blood glucose ≥ 7.8 mmol/L, regardless of the diabetic status. We calculated stress hyperglycemia ratio (SHR) with the following formula: FBG (mmol/L)/HbA1c-derived estimated average glucose, where HbA1c-derived estimated average glucose (mmol/L) = (1.59×HbA1c)-2.59 (19).

Besides, we also collected available capillary blood glucose levels during the first 36 hours after EVT. Considering the different frequency of measurement in each center, we mainly collected three specific times of each day: fasting (≥8 hours), postprandial (2 hours after the meal) and nighttime. During the monitoring period, various parameters of capillary glucose level were used to analyze the short-term GV: mean glucose level, standard deviation (SD) of the mean and coefficient of variation (CV) (8). The CV for glucose was calculated as [SD/mean glucose] × 100% (20).

### Outcome assessment

Safety outcomes included symptomatic intracranial hemorrhage (sICH) and in-hospital mortality. Intracranial hemorrhage was classified according to the Heidelberg Bleeding Classification within 48 hours after EVT. SICH was defined as a new intracranial hemorrhage detected by brain imaging, which was associated with an increase by ≥ 4 points of total NIHSS, or an increase by ≥ 2 points of a NIHSS subcategory, or neurological deterioration leading to major medical/surgical intervention (21).

Functional outcomes were assessed by the mRS score at 90 days and 1 year after stroke through routine telephone interview or face-to-face visit with patients or their relatives. Poor functional outcome was defined as mRS scores of 4-6.

### Statistical analysis

All statistical analyses were conducted with Stata 14.1, and a two-sided P value <0.05 was considered to be statistically significant.

Baseline characteristics, treatment profiles and outcomes were compared between patients with admission hyperglycemia and without admission hyperglycemia. Categorical variables were reported as number (percentages), and continuous variables were presented as mean ± standard deviation (SD) or median (interquartile range [IQR]) according to normality. The percentages of categorical variables were compared using the Pearson’s χ^2^ tests, and continuous variables were compared using the Student’s *t*-tests or the Wilcoxon rank sum test, as appropriate.

When studying the correlation between admission hyperglycemia, FBG and SHR and outcomes, we imputed 25 complete datasets with multivariate imputation by chained equations incorporating all analysis related variable. The imputation model contained 5 missing variables (GCS score, puncture to reperfusion time, admission hyperglycemia, FBG and HbA1c), of which the missing values of 3 variables are less than 10%, one variable is 10.9%, and one variable is 23.7%. We imputed continuous variables with linear regression and categorical variables with logistic regression. All estimates were obtained by averaging results across the 25 imputed datasets with inferences under multiple imputation obtained with Rubin’s rules. We also performed a sensitivity analysis on patients with complete data. Considering that only some patients had capillary blood glucose monitoring and the ratio of missing value was high, we used complete data to analyze the correlation between GV and outcomes.

To ascertain the independent contribution of admission hyperglycemia, FBG and SHR to outcomes (sICH and mRS), multivariable logistic regression analyses were performed and the prespecified covariates were adjusted: age, sex, hypertension, hyperlipidemia, baseline NIHSS, GCS, TOAST classification, baseline pc-ASPECT score, collateral status, BATMAN score, treatment with IVT, time from estimated occlusion to groin puncture, time from puncture to reperfusion, mTICI and previous DM. In exploring the association between GV and outcomes, we further adjusted for the use of tube feeding in patients.

Subgroup analyses were carried out to explore whether there are differences in the influence of blood glucose on the incidence of outcomes between diabetic subgroups. Interactions were examined by adding product terms to the logistic regression model to assess whether previous DM modified the association between glucose (admission hyperglycemia, FBG, SHR and GV) and outcomes.

In addition to the quantitative relationships, we used restricted cubic splines with 4 knots (at the 5th, 35th, 65th and 95th percentiles) to explore the nonlinear relationships between FBG, SHR and functional outcomes.

## Results

**Figure 1:**
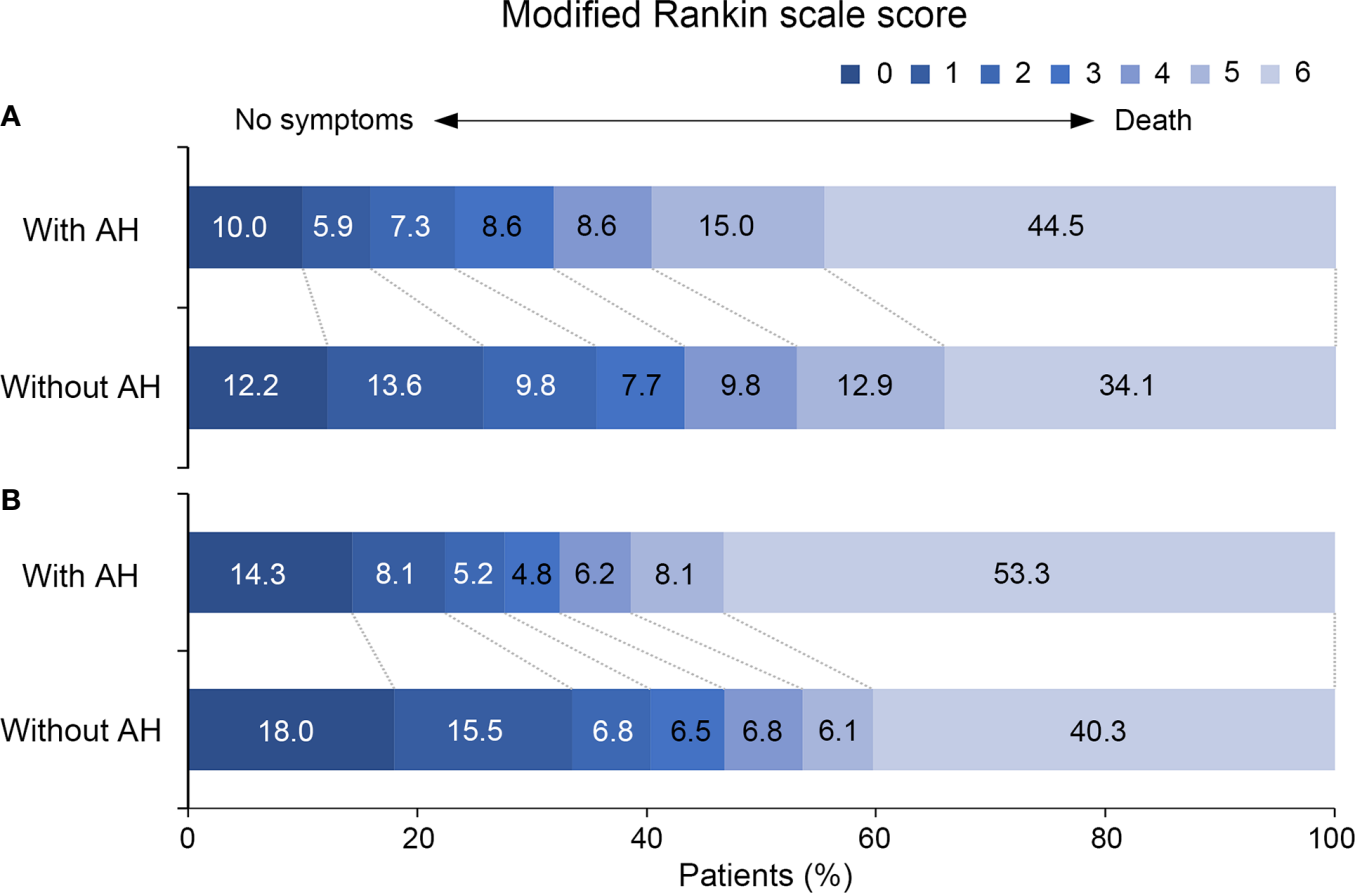
Distribution of the modified Rankin Scale Scores at **(A)** 90 days and **(B)** 1 year for patients with and without admission hyperglycemia. AH, admission hyperglycemia.

Out of a total 609 consecutive VBAO patients treated with EVT, we identified 569 eligible patients (**Figure I** in the **Data Supplement**). After one year follow up, 22 patients were lost. Details on the missing data can be found in **Figure II** in the **Data Supplement**. Baseline characteristics based on the presence of admission hyperglycemia are summarized in **Table 1**. Compared with patients without admission hyperglycemia, patients with admission hyperglycemia were more likely to have a higher prevalence of DM (p < 0.001) and lower BATMAN score (p = 0.040). No difference in the rates of successful recanalization (mTICI score 2b or 3) was detected between the two groups (85.5% vs. 84.7%, p = 0.806). Patients with admission hyperglycemia also had higher rates of sICH (11.4% vs. 3.8%, p = 0.001), in-hospital mortality (29.1% vs. 19.2%, p = 0.009) and poor functional outcomes at 90 days (68.2% vs. 56.8%, p = 0.009) and 1 year (67.6% vs. 53.2%, p = 0.001, **Table 1**). The distribution of mRS scores at 90 days and 1 year in patients with and without admission hyperglycemia was displayed in **Figure 1**.

**Table 1:** Baseline characteristics (n=507).

	Admission Hyperglycemia (n = 220)	No Admission Hyperglycemia (n = 287)	P
Age, mean ± SD, y	63.4 ± 12.1	64.0 ± 13.6	0.576
Male, n (%)	164 (74.5)	203 (70.7)	0.341
Hypertension, n (%)	158 (71.8)	183 (63.8)	0.055
Diabetes mellitus, n (%)	131 (59.5)	37 (12.9)	<0.001
Hyperlipidemia, n (%)	92 (41.8)	97 (33.8)	0.064
Previous stroke/TIA, n (%)	39 (17.7)	59 (20.6)	0.424
Coronary artery disease, n (%)	20 (9.1)	28 (9.8)	0.800
Smoking, n (%)	65 (29.5)	96 (33.4)	0.349
Baseline NIHSS, median (IQR)	22.0 (14.0-28.0)	23.0 (14.0-31.0)	0.459
Glasgow Coma Score, median (IQR), (n=506)	8 (6-11)	7 (6-12)	0.831
TOAST classification, n (%)			0.005
Large artery atherosclerosis	161 (73.2)	171 (59.2)	
Cardioembolism	35 (15.9)	67 (23.3)	
Others	24 (10.9)	50 (17.4)	
Baseline pc-ASPECT score, median (IQR)	9 (7-10)	9 (8-10)	0.163
Collateral status, n (%)			0.062
ASITN/SIR grade 0-1	173 (78.6)	214 (74.6)	
ASITN/SIR grade 2	36 (16.4)	42 (14.6)	
ASITN/SIR grade 3-4	11 (5.0)	31 (10.8)	
BATMAN score, median (IQR)	4 (3-6)	5 (3-7)	0.040
Onset patterns, n (%), (n=506)			0.139
Maximum neurological deficit from onset	97 (44.1)	143 (50.0)	
Progressive stroke	105 (47.7)	112 (39.2)	
Others	18 (8.2)	31 (10.8)	
Treatment with intravenous thrombolysis, n (%)	37 (16.8)	55 (19.2)	0.497
Time from estimated occlusion to groin puncture, median (IQR), min	332.5 (236.0-523.8)	330.0 (216.0-494.0)	0.280
Time from puncture to reperfusion, median (IQR), min, (n=476)	105.0 (76.0-165.0)	110.0 (70.0-150.0)	0.618
Type of initial application, n (%)			0.703
Stent retriever	176 (80.0)	232 (80.8)	
Aspiration	17 (7.7)	17 (5.9)	
Others	27 (12.3)	38 (13.2)	
mTICI score 2b or 3	188 (85.5)	243 (84.7)	0.806
sICH	25 (11.4)	11 (3.8)	0.001
In-hospital mortality	64 (29.1)	55 (19.2)	0.009
Poor functional outcome (mRS 4-6) at 90 days	150 (68.2)	163 (56.8)	0.009
Poor functional outcome (mRS 4-6) at 1 year (n=488)	142 (67.6)	148 (53.2)	0.001

SD, standard deviation; TIA, transient ischemic attack; NIHSS, National Institutes of Health Stroke Scale; IQR, interquartile range; TOAST, Trial of Org 10172 in Acute Stroke Treatment; pc-ASPECT score, posterior circulation-Alberta Stroke Program Early CT Score; ASITN/SIR, American Society of Interventional and Therapeutic Neuroradiology/Society of Interventional Radiology; BATMAN, Basilar Artery on Computed Tomography Angiography; mTICI, modified Thrombolysis in Cerebral Infarction; sICH, symptomatic intracranial hemorrhage; mRS, modified Rankin Scale.

### Perioperative glucose levels and outcomes

After adjustment for potential confounders, patients with admission hyperglycemia were more likely to have higher risk of sICH (odds ratio [OR] 3.24, 95% confidence interval [CI]: 1.40-7.46, p = 0.006, **Table 2**). Admission hyperglycemia was also independently associated with poor functional outcomes at 90 days and 1 year (OR 1.91, 95% CI: 1.15-3.18 for 90 days; OR 1.96, 95% CI: 1.20-3.22 for 1 year; **Table 2**). Multivariable analyses showed that increased FBG and SHR levels were significantly associated with worse outcomes, including sICH (OR 1.16, 95% CI: 1.05-1.30 for FBG; OR 3.53, 95% CI: 1.35-9.18 for SHR), poor functional outcomes at 90 days (OR 1.10, 95% CI: 1.02-1.20 for FBG; OR 3.06, 95% CI: 1.44-6.51 for SHR) and 1 year (OR 1.10, 95% CI: 1.02-1.19 for FBG; OR 3.04, 95% CI: 1.45-6.38 for SHR). Besides, the relationship between FBG levels and in-hospital mortality was also statistically significant (OR 1.08, 95% CI: 1.00-1.17, p = 0.037, **Table 2**).

**Table 2:** The associations between perioperative glucose levels and outcomes.

Outcomes		Unadjusted model		Adjusted model^*^	
		OR (95%CI)	P	OR (95%CI)	P
sICH	Admission hyperglycemia	2.86 (1.39-5.88)	0.004	3.24 (1.40-7.46)	0.006
	FBG	1.12 (1.03-1.21)	0.006	1.16 (1.05-1.30)	0.006
	SHR	3.47 (1.42-8.51)	0.007	3.53 (1.35-9.18)	0.010
In-hospital mortality	Admission hyperglycemia	1.67 (1.11-2.51)	0.014	1.55 (0.93-2.60)	0.092
	FBG	1.10 (1.04-1.16)	0.001	1.08 (1.00-1.17)	0.037
	SHR	1.84 (0.97-3.49)	0.063	1.70 (0.87-3.32)	0.118
Poor functional outcome (mRS 4-6) at 90 days	Admission hyperglycemia	1.71 (1.19-2.45)	0.004	1.91 (1.15-3.18)	0.012
	FBG	1.11 (1.05-1.18)	0.001	1.10 (1.02-1.20)	0.018
	SHR	3.37 (1.85-6.13)	<0.001	3.06 (1.44-6.51)	0.004
Poor functional outcome (mRS 4-6) at 1 year (n=547)	Admission hyperglycemia	1.78 (1.24-2.55)	0.002	1.96 (1.20-3.22)	0.008
	FBG	1.11 (1.05-1.18)	0.001	1.10 (1.02-1.19)	0.016
	SHR	3.21 (1.75-5.87)	<0.001	3.04 (1.45-6.38)	0.003

*Adjusted for age, sex, hypertension, hyperlipidemia, baseline NIHSS, Glasgow Coma Score, TOAST classification, Baseline pc-ASPECT score, Collateral status, BATMAN score, treatment with intravenous thrombolysis, time from estimated occlusion to groin puncture, time from puncture to reperfusion, mTICI and DM.

OR, odds ratio; CI, confidence interval; sICH, symptomatic intracranial hemorrhage; FBG, fasting blood glucose; SHR, stress hyperglycemia ratio; mRS, modified Rankin Scale.

**Figure 2:**
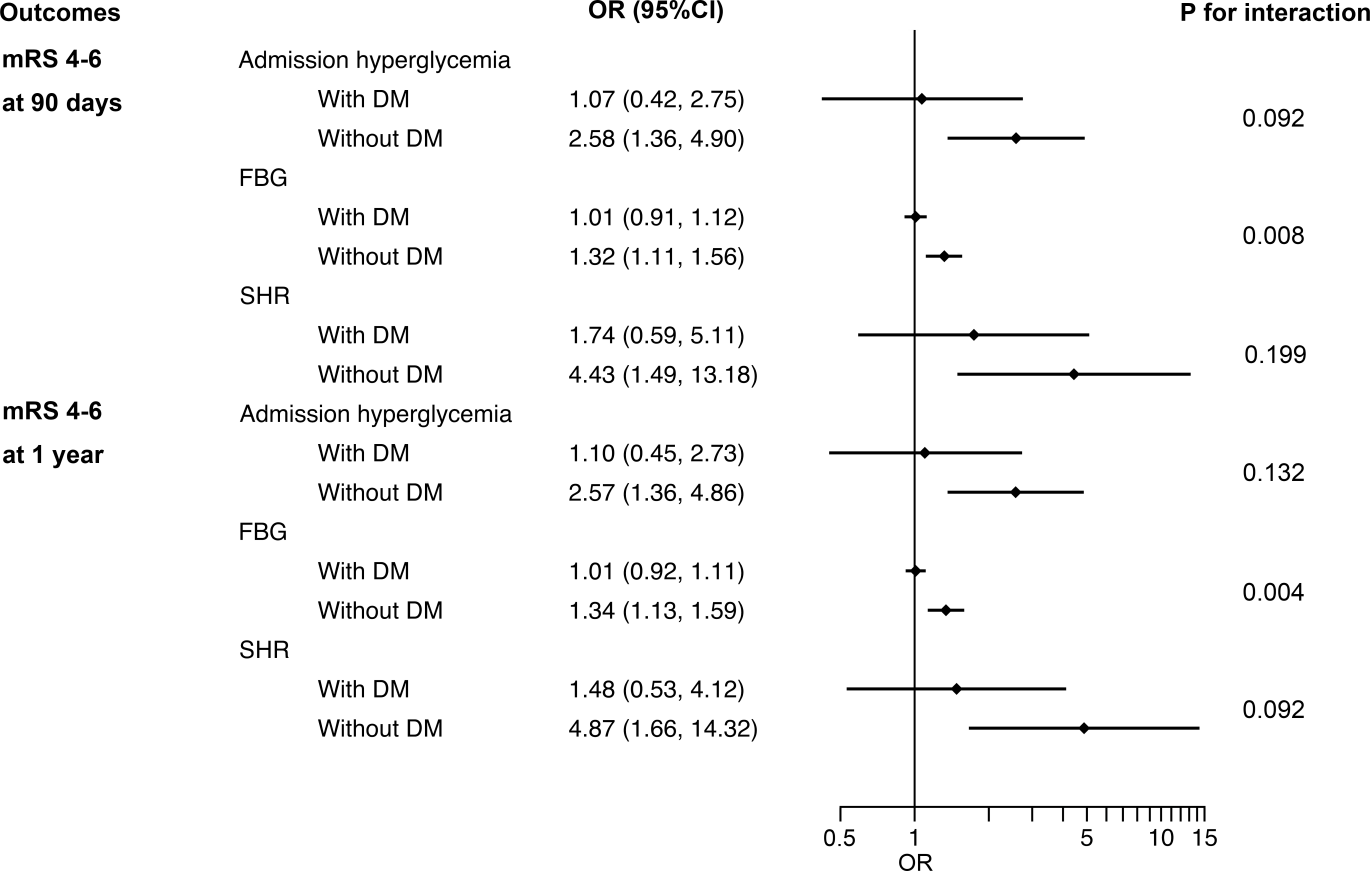
Interaction between DM and admission hyperglycemia, FBG and SHR on poor functional outcomes at 90 days and 1 year. DM, diabetes mellitus; FBG, fasting blood glucose; SHR, stress hyperglycemia ratio; OR, odds ratio; CI, confidence interval; mRS, modified Rankin Scale.

In subgroup analysis, the correlation between perioperative glucose levels and outcomes in non-diabetic patients was similar to that in the entire patients. Nevertheless, in patients with previous DM, no significant association was observed between admission hyperglycemia, FBG, SHR levels and outcomes (**Figure 2** and **Figure III** in the **Data Supplement**).

For outcome variables, such as sICH (p for interaction = 0.039), poor functional outcomes at 90 days (p for interaction = 0.008) and 1 year (p for interaction = 0.004), the interaction effect between previous DM and FBG was significant, indicating that the influence of FBG levels on these adverse outcomes in non-diabetic patients is greater than that in DM patients (**Figure 2** and **Figure III** in the **Data Supplement**). No evidence of interaction effects between admission hyperglycemia or SHR and previous DM was found regardless of the outcome variables. The same results were obtained by sensitivity analyses of patients with complete data (**Table I** in the **Data Supplement**).

**Figure 3:**
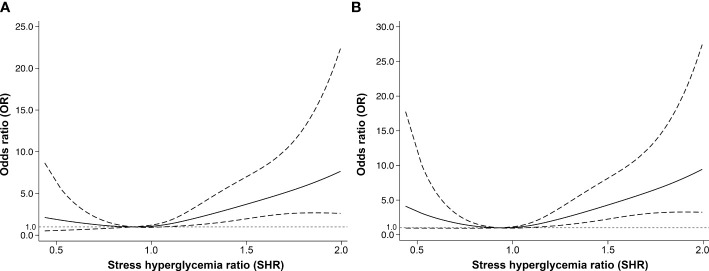
The nonlinear relationship between stress hyperglycemia ratio and adjusted odds ratios of poor functional outcomes at **(A)** 90 days and **(B)** 1 year. The nonlinear relationship was modeled by restricted cubic splines. The dashed lines indicate the 95% confidence intervals of the nonlinear solid line.

We observed a J-shaped association between SHR and the risk of poor functional outcomes at 90 days in multiple-adjusted restricted cubic spline regression, with the lowest point of SHR of 0.90, although not statistically significant (p for nonlinearity = 0.103, **Figure 3A**). There was also a nonlinear correlation between SHR and poor functional outcome at 1 year (p for nonlinearity = 0.010), showing a similar J-shaped curve (**Figure 3B**). That is, with the increase of SHR, the risk of poor functional outcome at 1 year first decreased and then increased. Although it can be observed that the risk of poor functional outcome increases with the increase of FBG, there is no nonlinear relationship between FBG and 90-day (p for nonlinearity = 0.056) or 1-year (p for nonlinearity = 0.132) poor functional outcomes (**Figure IV** in the **Data Supplement**).

### Short-term GV and outcomes

We obtained available capillary blood glucose levels of 266 patients within 36 hours after EVT (median number of measurements 9, IQR 8-11). The median CV of all patients was 18.25% (IQR 13.23-23.16). Patients with sICH had higher CV levels than those without sICH (19.58 [18.41-21.65] vs. 17.62 [13.05-23.23], p = 0.048). However, after adjusting for confounding factors, there was no significant correlation between CV and sICH (OR 1.04, 95% CI: 0.97-1.12, p = 0.275). While, the results of multivariable analysis showed that CV was independently associated with poor functional outcomes at 90 days (OR 1.23, 95% CI: 1.14-1.33, p < 0.001) and 1 year (OR 1.25, 95% CI: 1.15-1.37, p < 0.001).

The results of the subgroup analyses showed that whether in patients with or without previous DM, CV was always significantly associated with poor functional outcomes at 90 days and 1 year (**Table 3**). For 1-year poor functional outcome, the interaction effect between previous DM and CV was significant (p for interaction = 0.042), suggesting that CV levels had a greater impact on non-diabetic patients than DM patients.

**Table 3:** The associations between short-term GV and outcomes.

Outcomes	Subgroups	Unadjusted model		Adjusted model*		Interaction
		OR (95%CI)	P	OR (95%CI)	P	
sICH		1.04 (0.98-1.10)	0.175	1.04 (0.97-1.12)	0.275	0.567
	DM	1.05 (0.97-1.15)	0.210	1.10 (0.97-1.25)	0.140	
	Non-DM	1.03 (0.94-1.13)	0.553	1.03 (0.90-1.18)	0.678	
Poor functional outcome (mRS 4-6) at 90 days		1.16 (1.11-1.22)	<0.001	1.23 (1.14-1.33)	<0.001	0.323
	DM	1.17 (1.09-1.26)	<0.001	1.18 (1.08-1.30)	<0.001	
	Non-DM	1.23 (1.12-1.36)	<0.001	1.57 (1.20-2.05)	0.001	
Poor functional outcome (mRS 4-6) at 1 year		1.16 (1.11-1.22)	<0.001	1.25 (1.15-1.37)	<0.001	0.042
	DM	1.14 (1.07-1.22)	<0.001	1.16 (1.06-1.28)	0.002	
	Non-DM	1.30 (1.16-1.46)	<0.001	1.89 (1.30-2.74)	0.001	

*Adjusted for age, sex, hypertension, hyperlipidemia, baseline NIHSS, Glasgow Coma Score, TOAST classification, Baseline pc-ASPECT score, Collateral status, BATMAN score, treatment with intravenous thrombolysis, time from estimated occlusion to groin puncture, time from puncture to reperfusion, mTICI, DM and tube feeding.

GV, glycemic variability; OR, odds ratio; CI, confidence interval; sICH, symptomatic intracranial hemorrhage; DM, diabetes mellitus; mRS, modified Rankin Scale.

## Discussion

In this study of patients with VBAO treated with EVT, we observed the predictive significance of admission hyperglycemia, FBG and stress hyperglycemia on sICH, poor functional outcomes at 90 days and at 1 year. Furthermore, FBG was also associated with in-hospital mortality. The predictive significance still exists in non-diabetic patients, but not in DM patients. In addition, there was a significant interaction between FBG and previous DM, indicating that FBG had a greater impact on sICH and poor functional outcomes (90 days and 1 year) of non-diabetic patients than those with previous DM. Further analysis showed that SHR was J-shaped associated with the risk of poor functional outcome at 1 year in VBAO patients treated with EVT. Our results also suggested that short-term GV after EVT was independently associated with poor functional outcomes at 90 days and 1 year, whether in patients with or without previous DM.

In our study, the median admission glucose was 7.6 mmol/L (interquartile range, 6.7-9.3), which was higher than that of patients with ACS received EVT (6.6 [5.7-7.7] mmol/L) reported in previous studies (22). In addition, the probability of previous DM (34.1% vs. 16.1%) and admission hyperglycemia (43.3% vs. 30.3%) were also higher (4). These findings are consistent with previous studies in which patients with PCS are more likely to have previous DM and higher admission blood glucose levels than patients with ACS (9, 10).

Hyperglycemia was considered to impair the efficacy of IVT by lowering recanalization rates (23). However, previous studies showed no significant correlation between admission hyperglycemia and successful reperfusion after EVT in ACS (4, 22). Consistent with this, in our study, there was also no significant difference in the rate of successful reperfusion after EVT between patients with and without hyperglycemia (85.3% vs. 84.9%), suggesting that the worse outcomes in patients with hyperglycemia after EVT may not be the consequence of reduced recanalization rate, no matter in anterior or in posterior circulation stroke.

Our results are in agreement with the findings of previous studies conducted in patients with ACS treated with EVT, showing that increased admission glucose was associated with higher risk of sICH (24), increased in-hospital mortality and worse functional outcomes at 90 days (4, 7). EVT is currently the most effective way of recanalization of the occluded vessels, which can promote oxygen re-entry into the ischemic brain (22). Because oxygen promotes the formation of free radicals together with glucose, patients receiving EVT may have a greater exposure to redox-mediated effects related to admission blood glucose levels (12). Hyperglycemia could increase oxidative stress, exacerbate blood-brain barrier permeability after cerebral ischemia/reperfusion injury, and increase the risk of brain edema and hemorrhage transformation (25), resulting in increased infarct volume and greater neurological deficit (26).

Stress hyperglycemia has not been specifically defined and is usually restricted to patients without previous DM (27). We used SHR as a quantitative indicator of stress hyperglycemia, because relative hyperglycemia has been proved to be a better biomarker for critical illness than absolute hyperglycemia (19). A J-shaped association between SHR and the risk of poor functional outcome at 1 year was found in our study, which means that moderate stress is beneficial. Previous studies have demonstrated a J-shaped association between serum glucose and functional outcome in patients with ischemic stroke (28). Also, in patients with ACS treated with EVT, the probability of poor functional outcome was first decreased and then increased along with the increasing of the admission blood glucose (4). Although the nonlinear relationship between SHR and poor functional prognosis has not been reported in patients treated with EVT, some studies have shown that both relative hyperglycemia and hypoglycemia are associated with higher mortality at 90 days (29), which may indicate the existence of a J-shaped correlation.

Our results suggested that elevated short-term GV was independently associated with poor functional outcomes in patients with PCS undergoing EVT, but not with increased risk of sICH. In a recent study, consistent with the results of our study, no significant correlation between CV after successful recanalization with EVT and increased risk of sICH was found (8). Besides, there was also no significant association between CV and poor functional outcomes. Considering that most of the patients enrolled in that study are patients with anterior circulation occlusion (85.7%), it can be speculated that the effect of GV on the prognosis of patients with anterior and posterior circulation may be different.

Subgroup analyses showed that hyperglycemia was significantly associated with adverse outcomes in non-diabetic patients, but not in DM patients. A possible explanation is that DM patients may be more tolerant to blood glucose variability. When blood glucose levels fluctuate in a high range, non-diabetic patients are more likely to suffer from impaired immune defense and microvascular environment disorder (6, 30). Another possible explanation is that DM patients are more likely to receive glucose-lowering therapy, which may reduce the amount of glucose diffused into the brain, thereby reducing harmful metabolic changes in the brain (31). Therefore, high-quality studies on larger samples are needed to verify the results of this study.

Besides the typical limitations inherent in retrospective analysis, our study has additional limitations. First, some patients with diseases (such as hemoglobin disorders and severe hepatic disease) that may affect HbA1c measurements were excluded from all the analyses, so our results cannot be extrapolated to these patient populations. Second, information on antidiabetic medications, course of treatment and long-term blood glucose control for DM patients before the endovascular treatment was mostly incomplete, and the impact of these factors on outcomes cannot be fully evaluated despite we used SHR to quantify stress hyperglycemia to adjust blood glucose control over the past 8-12 weeks (32). Finally, because this was a retrospective registration trial, standard meals were not used, which may have affected the accuracy of CV calculations. Only a part of patients completed GV measurement, and the measurement duration is limited to a short time, so the interpretation of the results needs to be cautious.

## Conclusions

In summary, our findings indicate that admission hyperglycemia, FBG and stress hyperglycemia in VBAO patients treated with EVT were associated with adverse post-stroke outcomes both in the general population and in the non-diabetic subgroup, but not in the DM subgroup. The result also suggested that GV could be an appropriate clinical target to reduce the adverse effect of glucose fluctuation on prognosis. Since this is a retrospective observational study, our results should be interpreted with caution.

## Data availability statement

The raw data supporting the conclusions of this article will be made available by the authors, without undue reservation.

## Ethics statement

The study was reviewed and approved by the ethics committee of the First Affiliated Hospital of University of Science and Technology of China. Written informed consent for participation was not required for this study in accordance with the national legislation and the institutional requirements.

## Author contributions

MG, JF, JZ, and WS conceived and designed the study. JF, PX, LX, JW, ML, CL, GL, QC, DL, and LY acquired the data. MG, LX and JW analyzed the data. MG drafted the manuscript. JZ and WS revised the manuscript and approved the final version of the manuscript. All authors reviewed and approved the final manuscript.

## Funding

This study was supported in part by Anhui Provincial Natural Science Foundation (No. 2008085QH368), Fundamental Research Funds for the Central Universities (WK9110000056) and High-level Talents Innovation and Entrepreneurship Project of Quanzhou Science and Technology Bureau (No.2018C049R).

## Conflict of interest

The authors declare that the research was conducted in the absence of any commercial or financial relationships that could be construed as a potential conflict of interest.

The reviewer XK declared a shared affiliation with the authors MG, JZ to the handling editor at the time of review.

## Publisher’s note

All claims expressed in this article are solely those of the authors and do not necessarily represent those of their affiliated organizations, or those of the publisher, the editors and the reviewers. Any product that may be evaluated in this article, or claim that may be made by its manufacturer, is not guaranteed or endorsed by the publisher.
