# Supplementary material for: Effects of perioperative glycemic indicators on outcomes of endovascular treatment for vertebrobasilar artery occlusion
Source: Front Endocrinol (Lausanne). 2022 Oct 5;13:1000030. doi: 10.3389/fendo.2022.1000030 (PMC9581226; doi:10.3389/fendo.2022.1000030)
Supplement: Supplementary file 1 [file DataSheet_1.docx]

**Supplementary Materials**

**Contents:**

**Figure I.** Flow chart.

**Figure II.** Missing data.

**Figure III.** Interaction between DM and admission hyperglycemia, FBG and SHR on sICH and in-hospital mortality.

**Figure IV.** The nonlinear relationship between fasting blood glucose and adjusted odds ratios of poor functional outcome at (A) 90 days and (B) 1 year.

**Table I.** The associations between perioperative glucose levels and outcomes (sensitivity analysis on patients with complete data).

**Figure I.** Flow chart.


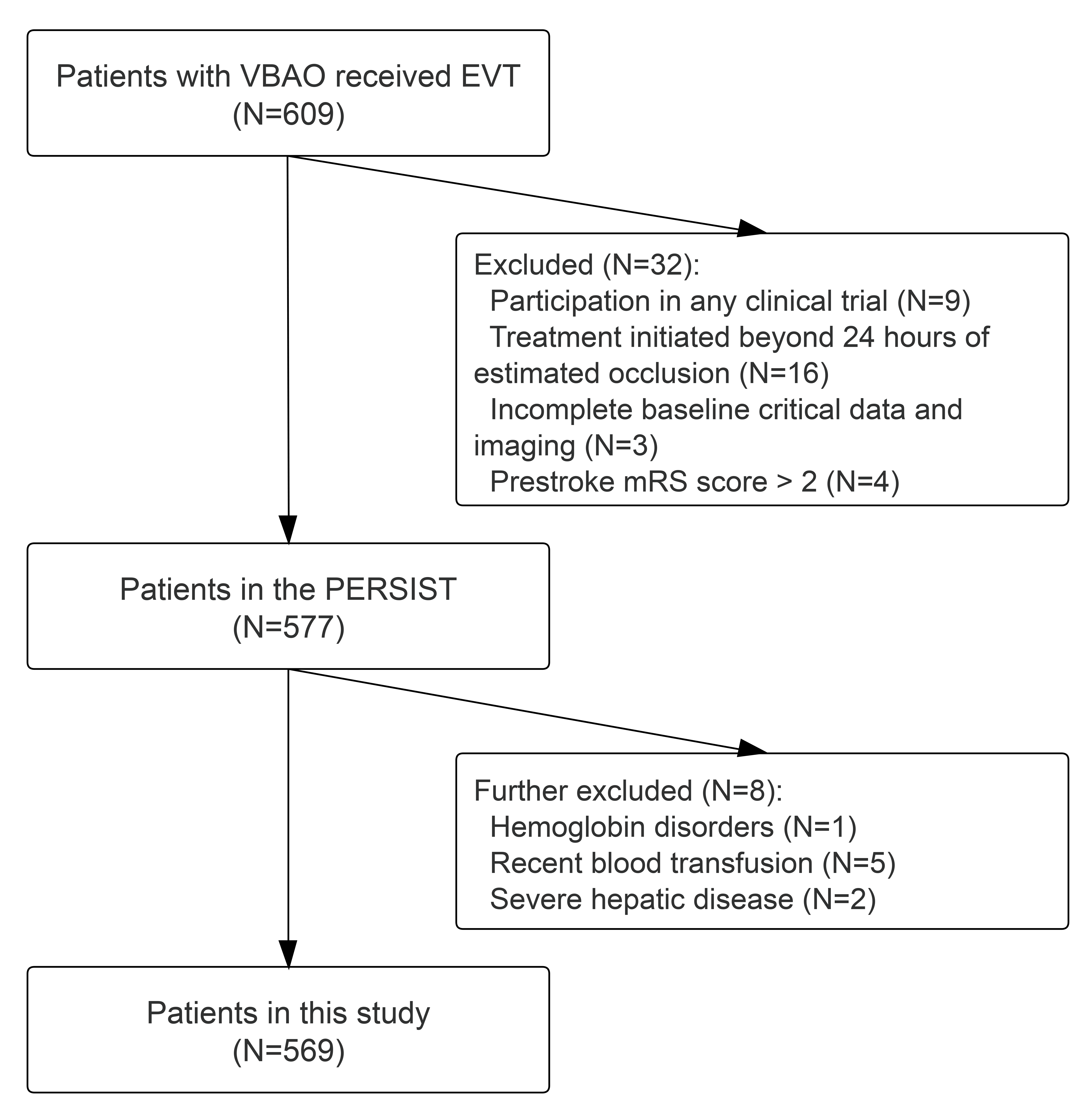


Abbreviations: VBAO = vertebrobasilar artery occlusion; EVT = endovascular treatment; mRS = modified Rankin scale; PERSIST = acute PostErior ciRculation iSchemIc Stroke registry.

**Figure II.** Missing data.


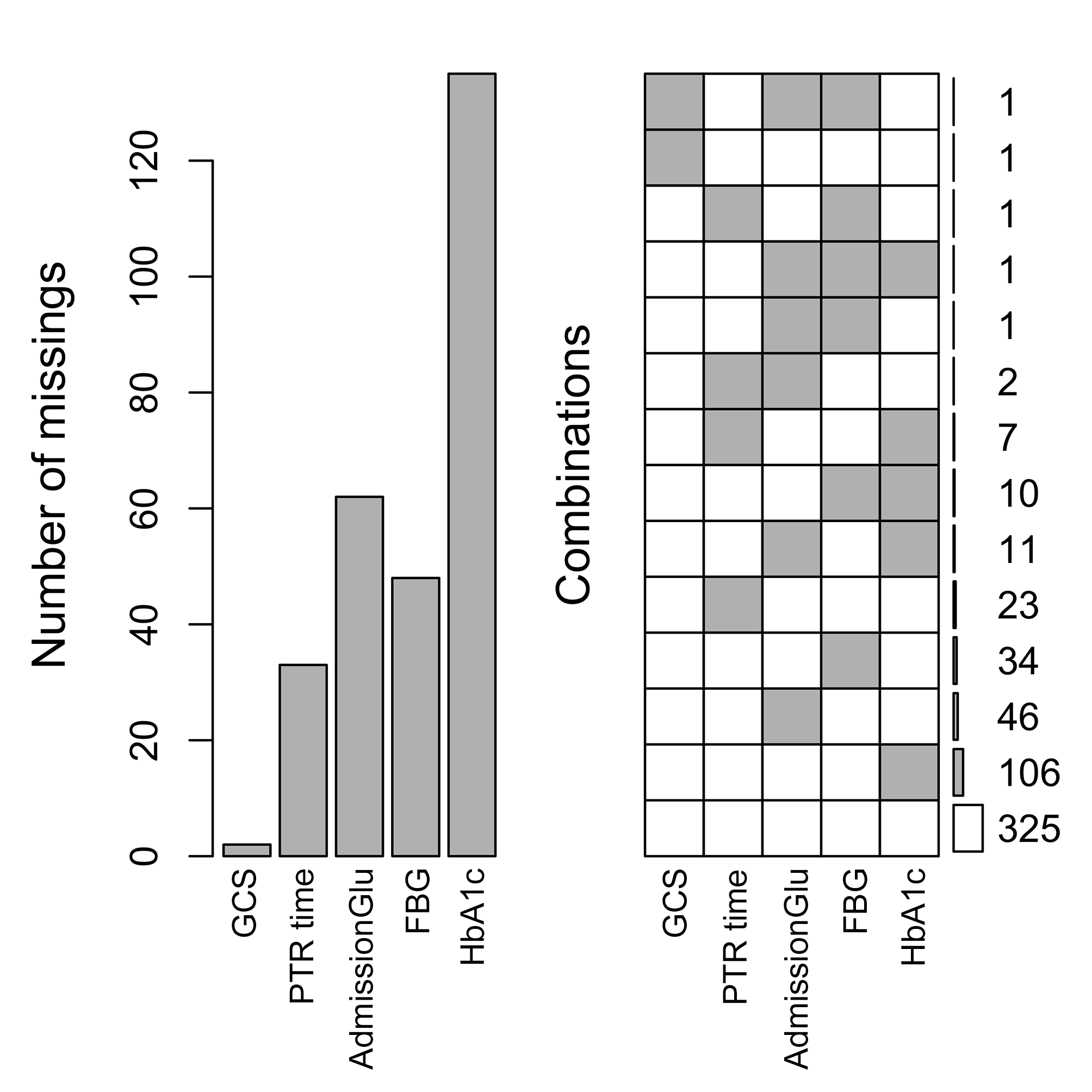


The gray values represent missing values.

Abbreviations: GCS = Glasgow Coma Scale; PTR time = puncture to reperfusion time; AdmissionGlu = admission glucose levels; FBG = fasting blood glucose; HbA1c = Hemoglobin A1c.

**Figure III.** Interaction between DM and admission hyperglycemia, FBG and SHR on sICH and in-hospital mortality.


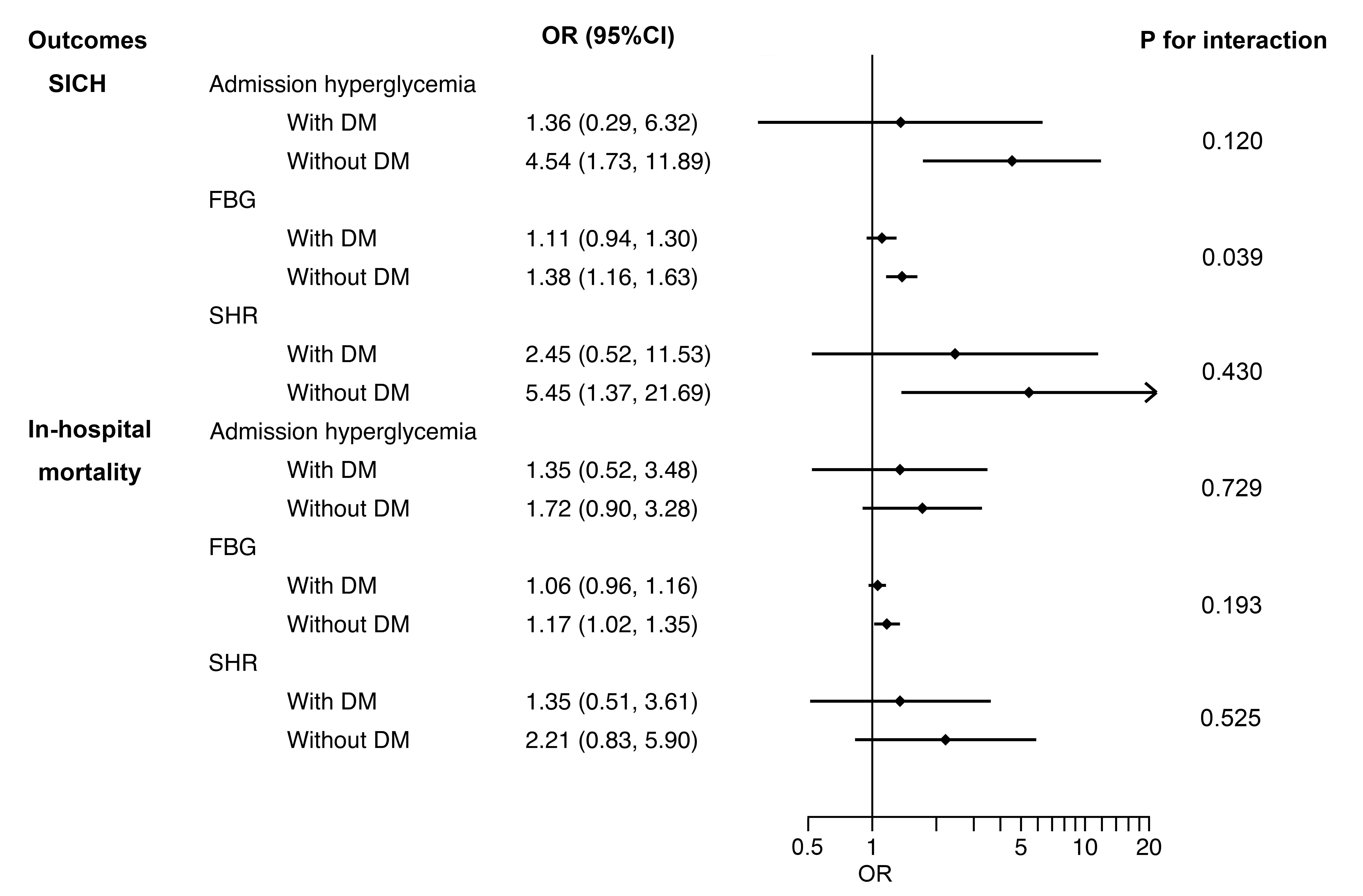


Abbreviations: DM = diabetes mellitus; FBG = fasting blood glucose; SHR = stress hyperglycemia ratio; OR = odds ratio; CI = confidence interval; sICH = symptomatic intracranial hemorrhage.

**Figure IV.** The nonlinear relationship between fasting blood glucose and adjusted odds ratios of poor functional outcome at (A) 90 days and (B) 1 year.


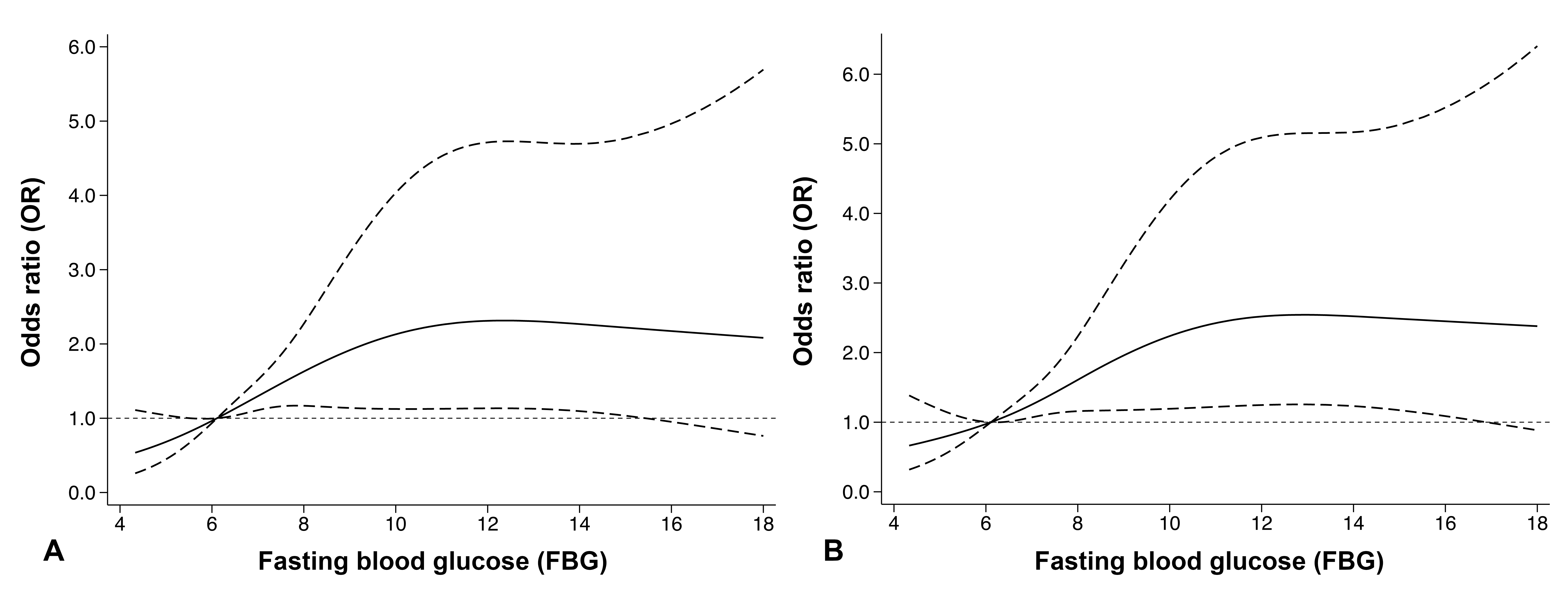


The nonlinear relationship was modeled by restricted cubic splines. The dashed lines indicate the 95% confidence intervals of the nonlinear solid line.

**Table I.** The associations between perioperative glucose levels and outcomes (sensitivity analysis on patients with complete data).

| Outcomes |  | All patients | | Without DM | | With DM | |
| --- | --- | --- | --- | --- | --- | --- | --- |
|  |  | OR (95%CI) | P | OR (95%CI) | P | OR (95%CI) | P |
| sICH | Admission hyperglycemia | 2.62 (1.09-6.26) | 0.030 | 3.76 (1.35-10.50) | 0.011 | 0.96 (0.19-4.79) | 0.962 |
|  | FBG | 1.14 (1.01-1.28) | 0.027 | 1.38 (1.14-1.68) | 0.001 | 1.06 (0.90-1.25) | 0.465 |
|  | SHR | 5.43 (1.90-15.54) | 0.002 | 21.62 (3.60-129.77) | 0.001 | 2.97 (0.52-17.11) | 0.223 |
| In-hospital mortality | Admission hyperglycemia | 1.39 (0.80-2.42) | 0.240 | 1.42 (0.71-2.85) | 0.320 | 1.40 (0.51-3.89) | 0.513 |
|  | FBG | 1.10 (1.02-1.19) | 0.018 | 1.17 (1.02-1.34) | 0.028 | 1.10 (0.99-1.22) | 0.066 |
|  | SHR | 1.93 (0.95-3.89) | 0.067 | 2.50 (0.92-6.83) | 0.074 | 2.14 (0.71-6.46) | 0.178 |
| Poor functional outcome (mRS 4-6) at 90 days | Admission hyperglycemia | 1.93 (1.14-3.28) | 0.015 | 2.63 (1.35-5.10) | 0.004 | 1.01 (0.35-2.90) | 0.979 |
|  | FBG | 1.12 (1.03-1.22) | 0.008 | 1.34 (1.13-1.59) | 0.001 | 1.04 (0.93-1.15) | 0.520 |
|  | SHR | 3.65 (1.72-7.75) | 0.001 | 7.23 (2.25-23.24) | 0.001 | 2.21 (0.72-6.80) | 0.167 |
| Poor functional outcome (mRS 4-6) at 1 year | Admission hyperglycemia | 2.17 (1.27-3.71) | 0.005 | 2.57 (1.33-4.97) | 0.005 | 1.29 (0.46-3.62) | 0.634 |
|  | FBG | 1.12 (1.03-1.22) | 0.007 | 1.36 (1.15-1.62) | <0.001 | 1.03 (0.93-1.14) | 0.551 |
|  | SHR | 3.39 (1.61-7.12) | 0.001 | 7.55 (2.32-24.60) | 0.001 | 1.82 (0.61-5.42) | 0.281 |

Adjusted for age, sex, hypertension, hyperlipidemia, baseline NIHSS, Glasgow Coma Score, TOAST classification, Baseline pc-ASPECT score, Collateral status, BATMAN score, treatment with intravenous thrombolysis, time from estimated occlusion to groin puncture, time from puncture to reperfusion, mTICI and DM.

Abbreviations: OR = odds ratio; CI = confidence interval; DM = diabetes mellitus; sICH = symptomatic intracranial hemorrhage; FBG = fasting blood glucose; SHR = stress hyperglycemia ratio; mRS = modified Rankin Scale.
